# Natural Conception After Tubal Reconstruction: A Rare Success Following Bilateral Fimbrioplasty for Post-pelvic Inflammatory Disease Occlusion

**DOI:** 10.7759/cureus.100262

**Published:** 2025-12-28

**Authors:** Swati Kumari, Sebastian Reyes Lizaola, Tajudeen Dabiri, Pengfei Wang

**Affiliations:** 1 Obstetrics and Gynecology, BronxCare Health System, New York, USA; 2 Obstetrics and Gynecology, Icahn School of Medicine at Mount Sinai, New York, USA

**Keywords:** fimbrioplasty, hydrosalpinx, infertility, natural conception, pelvic inflammatory disease, reproductive surgery, robotic surgery, tubal infertility

## Abstract

Pelvic inflammatory disease (PID) remains a major cause of tubal factor infertility. Although in vitro fertilization (IVF) has largely replaced reconstructive surgery, selected patients with mild tubal damage may still achieve natural conception after surgical repair. This case highlights a spontaneous conception following bilateral fimbrioplasty in a patient with mild post-PID distal tubal occlusion.

A 33-year-old, gravida 4 para 2, woman presented with three years of secondary infertility. Hysterosalpingogram (HSG) showed mild bilateral hydrosalpinx and distal tubal occlusion, with normal ovarian reserve and hormonal evaluation. The patient underwent robotic fimbrioplasty on July 23, 2025. Operative findings included Fitz-Hugh-Curtis changes, bilateral filmy adhesions, and grade-I distal occlusion. Bilateral adhesiolysis and fimbrioplasty were performed with restoration of tubal patency on chromotubation. The postoperative course was uncomplicated. Ten weeks after surgery, the patient conceived spontaneously with a normal intrauterine pregnancy. Tubal reconstructive surgery remains a valuable fertility-preserving option for selected patients who decline or lack access to IVF. Success depends on careful patient selection, minimal tubal damage, and meticulous microsurgical technique.

Tubal reconstruction can offer selected women an affordable, fertility-preserving option, especially in low-resource settings where IVF is inaccessible.

## Introduction

Tubal factor infertility continues to be one of the major causes of female infertility, accounting for nearly one-third of all cases [1]. The distal fimbrial portion of the fallopian tube is particularly vulnerable to damage from pelvic inflammatory disease (PID), endometriosis, or prior pelvic surgery [2]. Hydrosalpinx formation due to distal blockage can impair fertility by exerting a toxic effect on embryos and lowering pregnancy rates, even with in vitro fertilization (IVF) [3]. Although IVF is now widely available, surgical correction of tubal pathology retains importance [4]. Laparoscopic and robotic fimbrioplasty and neosalpingostomy are well-established techniques to restore tubal patency and preserve natural fertility [5]. Reported pregnancy rates vary from 25% to over 60%, depending on patient selection and disease severity [6]. This report presents a 33-year-old woman with bilateral distal tubal occlusion secondary to PID who conceived naturally following bilateral fimbrioplasty. No intraoperative photographs were available because images were not obtained during the procedure; however, all operative findings were documented in detail. This case is reported to highlight the continued clinical relevance of reconstructive surgery as a cost-effective alternative to IVF.

## Case presentation

A 33-year-old gravida 4 para 2 with zero preterm births, one abortion, and two living children (G4P2012) woman presented with three years of secondary infertility. She had two prior spontaneous vaginal deliveries (2012, 2018) and one miscarriage at eight weeks managed with dilation and curettage. Following her last delivery, the patient reported using barrier methods for contraception. She reported a treated episode of PID approximately four years prior to presentation, which likely contributed to the tubal factor infertility. She had no chronic medical conditions. Laboratory evaluation showed normal ovarian reserve, with follicle-stimulating hormone (FSH) 5.7 mIU/mL and estradiol 64.8 pg/mL on day 3, prolactin 4.15 ng/mL, thyroid-stimulating hormone (TSH) 0.97 µIU/mL, hemoglobin (HbA1c) 5.7%, and vitamin D 23.2 ng/mL. Ovulation was confirmed via a history of regular monthly menstrual cycles. Anti-Müllerian hormone (AMH) and antral follicular count (AFC) were not obtained, given the normal day 3 FSH and regular menstruation. Hemoglobin electrophoresis revealed sickle cell trait. Infectious disease screening, including human immunodeficiency virus (HIV), rapid plasma reagin (RPR), hepatitis B surface antigen (HBsAg), and hepatitis C virus (HCV) testing, was negative (Table 1).

**Table 1:** Laboratory investigations FSH: follicle-stimulating hormone; LH: luteinizing hormone; TSH: thyroid-stimulating hormone; HbA1c: hemoglobin; HIV: human immunodeficiency virus; RPR: rapid plasma reagin

Parameter	Patient Value	Reference Range
FSH (day 3)	5.7 mIU/mL	Follicular 3-10 mIU/mL
LH (day 3)	64.8 pg/mL	Follicular <80 pg/mL
Prolactin	4.15ng/mL	4-23 ng/mL
TSH	0.97 microIU/mL	0.4-4.5 microIU/mL
HbA1c	5.70%	<5.7%; normal 5.7-6.4% (prediabetes)
Vitamin D	23.2 ng/mL	30-100 ng/mL
Hemoglobin electrophoresis	Sickle cell trait	Normal AA pattern
HIV	Negative	Negative
RPR	Non-reactive	Non-reactive
Hepatitis surface antigen	Negative	Negative
Hepatitis C	Negative	Negative

**Figure 1:**
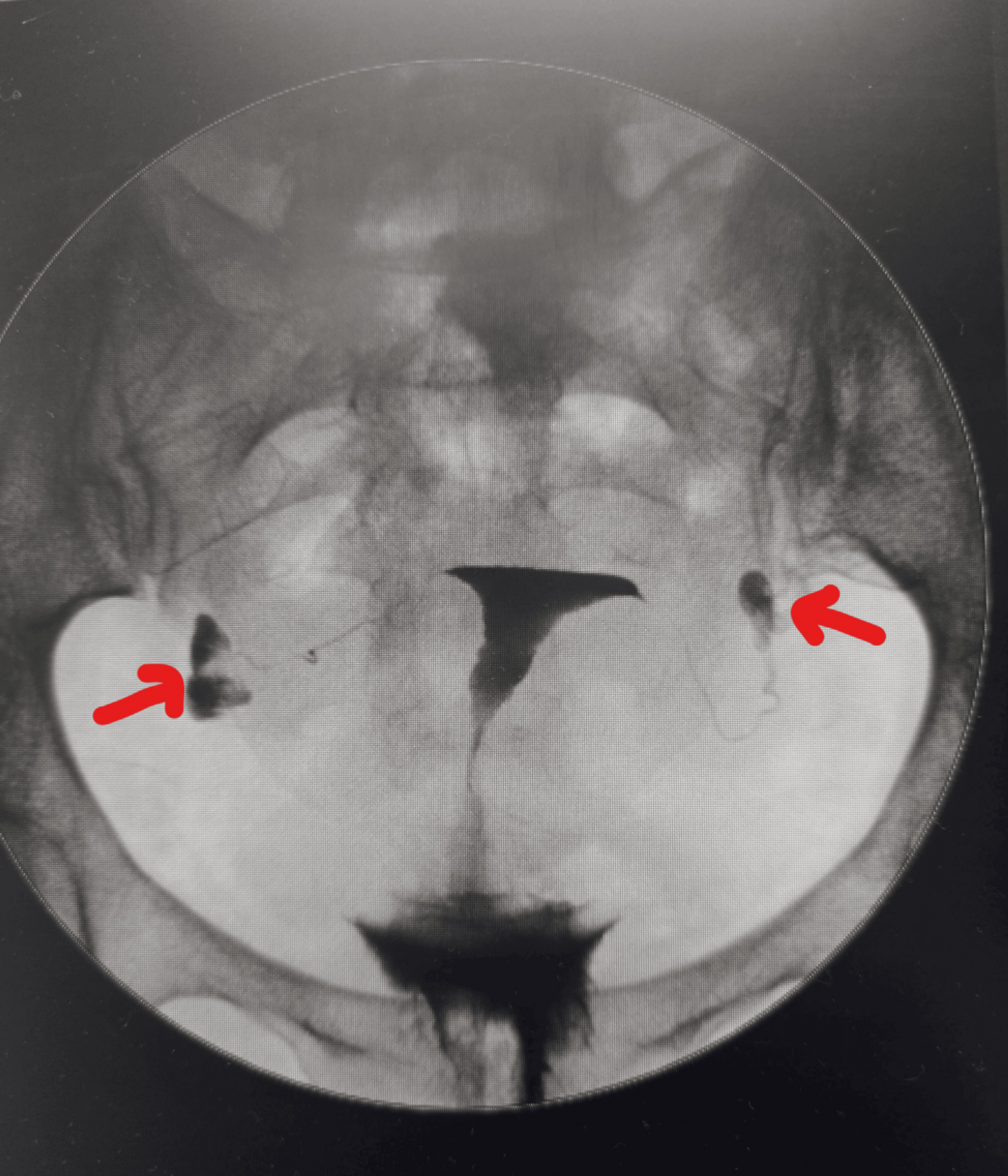
Hysterosalpingogram showing distal hydrosalpinx (arrows)

Ultrasound was normal. Hysterosalpingogram (HSG) demonstrated bilateral distal occlusion with mild hydrosalpinx (Figure 1).

**Figure 2:**
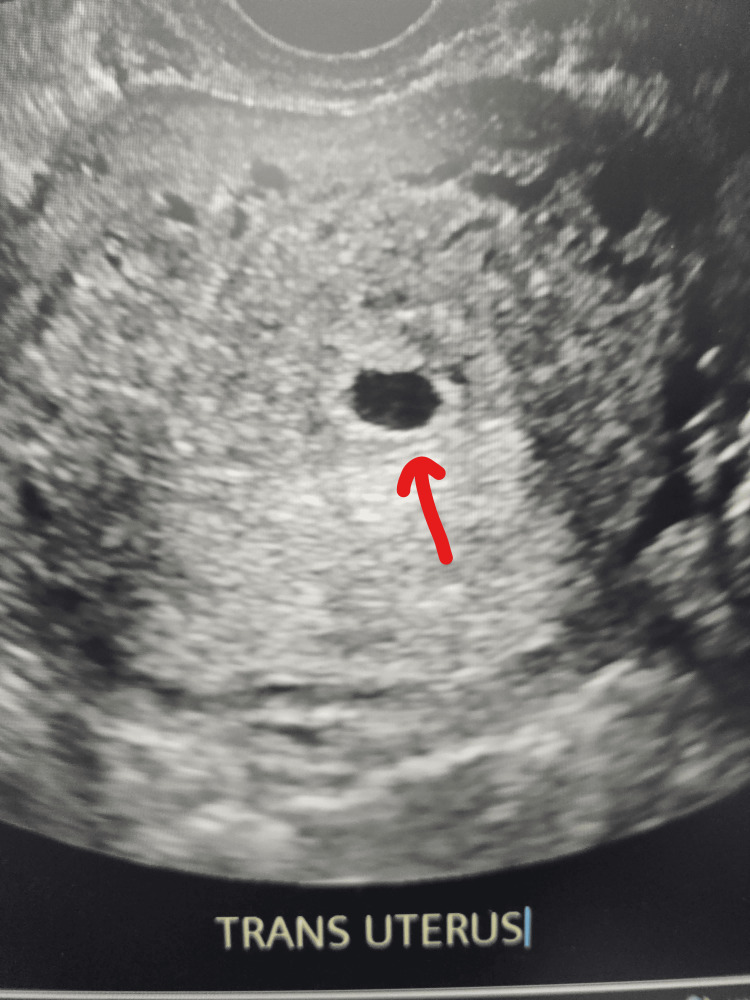
Ultrasonogram showing early intrauterine pregnancy (arrow)

Given bilateral distal disease and cost-prohibitive IVF, she elected robotic bilateral fimbrioplasty and adhesiolysis. Intraoperatively, Fitz-Hugh-Curtis adhesions and bilateral filmy peritubal adhesions were noted. Peritubal adhesions were excised sharply. Chromotubation showed patency on the left but not the right. Right fimbrioplasty was performed by creating three flaps (1, 6, 11 o'clock) and suturing them to the serosa using 2-0 Vicryl (Ethicon, Somerville, NJ, USA). Patency was confirmed bilaterally on repeat chromotubation. Intraoperative images were not available for inclusion, as they were not routinely captured at the time of surgery. The patient was discharged on the same day and advised timed intercourse. Ten weeks post-surgery, she conceived spontaneously, and an early intrauterine pregnancy was confirmed (Figure 2).

## Discussion

The management of distal tubal occlusion involves a key decision - whether to perform reconstructive surgery or proceed directly to IVF. The high surface area and delicate ciliated epithelium of the fimbriae make them particularly susceptible to inflammatory damage and agglutination following pelvic infection. Evidence-based reviews, including a Cochrane analysis, support considering tubal reconstructive surgery in well-selected patients with mild to moderate disease, good ovarian reserve, and normal semen analysis in their partners [4]. In contrast, severe tubal damage, dense adhesions, or gross hydrosalpinx often favor IVF due to lower surgical success rates [5,6]. However, IVF remains financially and geographically inaccessible to many women worldwide, especially in underserved communities [7-9]. Recent studies continue to support the careful selection of patients for tubal reconstructive procedures [10]. Moreover, cost-effectiveness analyses have demonstrated that for women under 35 with good ovarian reserve, surgical repair may offer a more affordable first-line approach, particularly in healthcare systems with limited IVF accessibility [11]. In this regard, patient-centered counseling that includes both financial and emotional considerations is crucial in guiding fertility management. In our case, the patient has no access to IVF financially, and surgical intervention was the only possible option for her to conceive another pregnancy. After evaluation, our patient was revealed to be an excellent candidate for tubal reconstruction surgery. The patient has a normal egg reserve with no significant comorbidities. The tubal disease was localized to the distal portion and manifested as mild hydrosalpinx. The uterus was non-remarkable. The semen analysis was not checked, but she had two normal pregnancies with her husband.

Published data suggest that pregnancy rates after fimbrioplasty or neosalpingostomy range from 30% to 60%, with patency rates exceeding 70% [6,8]. In one large series of 402 laparoscopic procedures, conception rates were highest among women with mild disease and decreased sharply with severe fibrosis or hydrosalpinx [2]. Similarly, Audebert et al. reported a 72% restoration of tubal patency and a 35% pregnancy rate after fimbrioplasty in a smaller cohort [9]. The most important predictors of success include the degree of tubal damage, the presence of healthy fimbrial mucosa, and minimal adhesions [5,7]. Older age, extensive fibrosis, and recurrent pelvic infections reduce the likelihood of success. In our case, clearly the tubal disease was secondary to PID due to the surgical findings, although she denies a history of sexually transmitted diseases. However, the patient’s tubes showed filmy adhesions rather than dense scarring, the presence of overall fimbriated mucosa tissues, and mild hydrosalpinx - all positive prognostic findings. The main risk following fimbrioplasty is ectopic pregnancy, reported in about 5-10% of cases [5,8]. This occurs because the repaired tube may not fully regain normal ciliary motion or peristaltic function. Therefore, early ultrasound confirmation of intrauterine pregnancy is critical in these patients. In this case, early follow-up imaging showed an intrauterine gestation, ruling out ectopic pregnancy. Recurrence of adhesions or reocclusion is another limitation. Even technically successful surgery can lose patency over time, especially in patients with previous PID [7,8]. For that reason, natural conception is most likely to occur within the first six to 12 months after surgery [5]. Our patient achieved a spontaneous pregnancy in three months after surgery. We attribute the rapid conception to the meticulous microsurgical technique, specifically the minimal use of thermal energy near the fimbria and precise mucosal alignment, which preserved ciliary function.

Microsurgical principles remain the cornerstone of successful tubal reconstruction. The advent of robotic-assisted microsurgery has redefined the precision and ergonomics of tubal reconstructive techniques. Enhanced 3D visualization, improved dexterity, and tremor filtration enable meticulous dissection and atraumatic handling of delicate fimbrial tissue [12].

While robotic assistance offers improved precision and ergonomics, current literature suggests that postoperative recovery and fertility outcomes are generally comparable to conventional laparoscopy [12]. As surgical technology continues to evolve, robotic fimbrioplasty may play a larger role in fertility preservation for appropriately selected women, especially when IVF resources are constrained. This underscores the importance of individualized reproductive planning informed by both clinical evidence and patient values.

## Conclusions

This case demonstrates that tubal reconstructive surgery remains an important fertility option. For well-selected patients, it offers the possibility of natural conception without the financial or emotional burden of IVF. While robotic platforms represent a high initial cost, the principle of tubal reconstruction (whether robotic or laparoscopic) offers a one-time intervention that can be more cost-effective than repeated IVF cycles in specific healthcare settings.
